# Living With COPD and Views on Digital Health Tools Among Pacific Peoples and Family Carers in Aotearoa New Zealand: A Qualitative Study

**DOI:** 10.1111/hex.70768

**Published:** 2026-07-14

**Authors:** Amio Matenga‐Ikihele, Joshua Ali'ifa'alogo, Michaela Roberts, Amy Chan, J. Geoffrey Chase, Ella F. S. Guy

**Affiliations:** ^1^ Moana Connect Auckland New Zealand; ^2^ Pacific Health Section School of Population Health, Faculty of Medical and Health Sciences, The University of Auckland Auckland New Zealand; ^3^ School of Pharmacy, Faculty of Medical and Health Sciences, University of Auckland Auckland New Zealand; ^4^ Faculty of Engineering Mechanical Engineering, University of Canterbury Christchurch New Zealand

**Keywords:** COPD, digital health technologies, digital inclusion, Pacific, respiratory health

## Abstract

**Background:**

Chronic Obstructive Pulmonary Disease (COPD) disproportionately affects Pacific peoples in Aotearoa New Zealand, with higher incidence, prevalence, and hospitalisation rates than non‐Māori, non‐Pacific populations. Despite this inequity, little qualitative research has explored how Pacific peoples and their families experience living with COPD, or their perspectives on emerging digital health technologies.

**Methods:**

This study adopted a qualitative design informed by *talanoa* methodology to explore the experiences of Pacific peoples living with COPD and their family caregivers. Participants were purposively recruited through Pacific community and professional networks. Talanoa sessions were audio‐recorded, transcribed verbatim, and analysed using a general inductive approach to identify key themes and subthemes.

**Results:**

Three interconnected themes were identified: (1) living with COPD, characterised by delayed diagnosis, constant vigilance, and repeated hospitalisation; (2) the central role of family in COPD management, with family members acting as caregivers and clinical partners within culturally grounded, collective systems of care; and (3) attitudes toward digital health tools. Participants expressed openness to digital health technologies to support COPD management, provided these tools were simple, reliable, culturally accessible, and designed to complement, not replace existing self‐management and family‐based care practices.

**Conclusion:**

Living with COPD for Pacific peoples is experienced as a collective and relational journey shaped by family, cultural values, and caregiving obligations, extending beyond individualised models of COPD self‐management. Family members play essential roles in managing COPD, yet this labour often remains unrecognised within formal health systems. Digital health tools offer potential to support COPD care if they are co‐designed with Pacific communities, align with Pacific values, and integrate within existing clinical and community care pathways. These findings highlight the need for culturally grounded, family‐inclusive approaches to COPD services and digital health innovation to advance health equity for Pacific peoples.

**Patient or Public Contribution:**

Patients living with COPD and family caregivers were central to this study as participants, contributing their lived experiences and perspectives. The study was guided by a Pacific relational approach that centred participant voices and the role of family in COPD care. Patients and the public were not involved in drafting the manuscript.

## 1 Introduction

Chronic Obstructive Pulmonary Disease (COPD) is a progressive, life‐limiting respiratory condition and a leading cause of morbidity and mortality worldwide [1]. While clinical management of COPD has advanced, Indigenous populations continue to experience a disproportionate burden of disease, characterised by higher prevalence, earlier onset, increased hospitalisation, and poorer outcomes compared with non‐Indigenous populations [2]. International research with Indigenous communities, including Aboriginal and Torres Strait Islander peoples in Australia, Māori in Aotearoa New Zealand, and First Nations peoples in Canada, consistently demonstrates that these inequities are shaped not only by smoking and environmental exposure, but by the enduring impacts of colonisation, structural racism, socioeconomic disadvantage, and limited access to culturally safe health care [2].

In Aotearoa New Zealand, COPD is the fourth leading cause of death, with an estimated 15% of adults aged 45 and over living with the condition [3]. The burden of COPD however is not distributed equally across the population. Pacific communities experience a markedly disproportionate impact, with incidence rates 2.1 times higher, prevalence 2.6 times higher, and hospitalisation rates 2.3 times higher than those of non‐Māori, non‐Pacific populations [3]. This disparity is further compounded by socioeconomic inequity. COPD follows a clear deprivation gradient, with higher rates of prevalence, hospitalisation, and mortality concentrated in the most deprived communities, contexts in which Pacific peoples are significantly overrepresented [4].

Understanding the scope of this inequity requires recognising who Pacific communities in Aotearoa New Zealand are. As a collective term, ‘Pacific communities’ refers to people with ancestral links to Pacific Island nations who now live in New Zealand, including Samoan, Tongan, Cook Islands Māori, Niuean, Tokelauan, and Fijian peoples, alongside smaller groups from Kiribati, Tuvalu, Papua New Guinea, the Solomon Islands, and French Polynesia [5]. There are over 18 Pacific ethnic groups residing in Aotearoa New Zealand. Importantly, they are not a homogeneous group. Pacific peoples represent a culturally rich and diverse population, shaped by distinct migration histories, island origins, languages, cultural practices and traditions. Recognising this diversity is essential to understanding the heterogeneous social, cultural, and structural determinants that collectively shape Pacific health outcomes in Aotearoa New Zealand [5].

Digital health technologies have been promoted internationally as a means of improving COPD management through remote monitoring, telehealth, and self‐management support [6]. Digital health encompasses the use of technologies, including mobile applications, remote monitoring devices, wearables, and virtual care platforms, to enhance health outcomes, support self‐management, and enable person‐centred care [7]. Critically, digital health technologies should benefit people in ways that are ethical, equitable, and sustainable, and be developed with principles of accessibility, privacy, and security [7]. For the purposes of this study, digital health refers specifically to mHealth technologies such as smartphone applications for symptom tracking and medication management, and remote physiological monitoring tools, which have been identified as relevant to COPD self‐management in community settings. While such tools show promise, evidence from Indigenous and marginalised populations cautions that digital health interventions may exacerbate inequities if they are not culturally grounded, accessible, and integrated within existing care practices [2, 8].

Despite growing international research with Indigenous populations, there remains limited qualitative evidence exploring how Pacific peoples experience COPD in Aotearoa New Zealand, particularly from the combined perspectives of individuals living with COPD and their family caregivers. This study addresses this gap by exploring Pacific patient and caregiver experiences of living with COPD, their approaches to management and care, and their attitudes towards using digital health technologies. By centring Pacific voices, this research aims to inform culturally grounded COPD services and guide the development of digital health tools that align with Pacific values, strengthen family‐based care, and contribute to more equitable respiratory health outcomes.

## 2 Methods

### 2.1 Study Design

This study used a qualitative design informed by *talanoa* methodology to explore the lived experiences of Pacific peoples with Chronic Obstructive Pulmonary Disease (COPD) and the family members involved in their care. The research examined participants' views on current COPD management and their attitudes toward the potential use of digital tools to support ongoing monitoring. The study was led by Pacific researchers with personal and professional connections to Pacific communities in Aotearoa New Zealand. This positionality informed the research throughout, shaping how *talanoa* sessions were facilitated, how participants were engaged, and how data were interpreted.

*Talanoa* was used as the primary research method. As an Indigenous storytelling practice embraced by numerous Pacific communities [9], *talanoa* provides a culturally grounded approach to dialogue and knowledge sharing. The term *talanoa* is derived from *tala*, to tell or narrate, and *noa*, meaning without concealment, reflecting the methodology's commitment to open and honest exchange [9, 10]. As an Indigenous Pacific qualitative approach grounded in relational dialogue and storytelling, *talanoa* reduces distance between researcher and participant and supports open, empathetic conversation [10].

Vaioleti describes *talanoa* as a relational encounter, ‘individually or in a group, made possible only by a desire by all involved to engage verbally, intellectually, spiritually even emotionally about issues at hand,’^(p. 128)^ highlighting its emphasis on genuine relational connection. Its flexible, conversational style differs from structured Western interview formats and enables participants to share experiences in ways that reflect core Pacific cultural values such as relationality, reciprocity, respect, and collective wellbeing [11].

### 2.2 Participants and Recruitment

Participants were recruited using purposive sampling and were eligible if they identified as Pacific peoples residing in Aotearoa New Zealand, were aged 18 years or older, and either had a physician‐diagnosis of COPD or were a family member or caregiver of someone living with COPD. Recruitment occurred through the research team's professional and community networks affiliated with Moana Connect. Recruitment materials were disseminated via email, radio, in‐person engagement, and promoted on Moana Connects social media platforms. Although recruitment spanned community, professional, and digital networks, response rates were modest, and enrolment concluded at 11 participants upon reaching thematic sufficiency, with no new insights emerging across later *talanoa*. Ethical approval was obtained from the University of Canterbury Human Research Ethics Committee (Reference: HREC 2025/38). All participants provided informed consent prior to participation, with options to consult family members before confirming involvement.

### 2.3 Data Collection

Data were collected through *talanoa* sessions conducted between October 2025 and February 2026, either in person, online via Microsoft Teams or Zoom or over the phone. Each *talanoa* was facilitated by two Pacific researchers and lasted between 30 and 60 min. The study comprised four one‐on‐one *talanoa*, two *talanoa* with pairs of family members, and one *talanoa* involving three family members. All *talanoa* were audio‐recorded with participant consent, or detailed notes were taken where recording was not preferred. Prior to commencing each *talanoa*, participants were provided with a participant information sheet and consent form. Written consent was obtained for in‐person sessions, while verbal consent was recorded for online sessions. As an acknowledgement of their contribution, participants received a supermarket voucher valued at $100 after each *talanoa*.

### 2.4 Data Analysis

Each *talanoa* was transcribed verbatim, de‐identified, and assigned unique participant codes. Data were analysed using a general inductive approach, led by two Pacific researchers [12]. Analysis began with close, repeated reading of transcripts alongside a replay of audio recordings to ensure fidelity to participants' words, context and intent. Coding was conducted collaboratively by both researchers, with regular sense‐making sessions used to discuss interpretive questions, work through areas of uncertainty, and refine emerging themes. As themes were developed iteratively, definitions were progressively clarified; transcripts were further analysed, and the data were revised and recoded to fewer categories. Thematic sufficiency was assessed across the dataset, with sufficiency considered reached when new talanoa data no longer generated new themes or meaningfully expanded existing ones. Final themes were confirmed through consensus within the research team.

## 3 Results

This study included 11 Pacific participants: five individuals living with COPD and six family members involved in their care. Most participants were within the 45–64 and 65+ age groups. Seven participants were female and four were male, with nine identifying as Samoan and two from Niue. Half of the caregivers were spouses, with others including parents and an adult daughter. An overview of participant demographics is presented in Table 1.

**Table 1:** Demographic characteristics of participants (*n* = 11).

Pseudonym (i.e. not real names)	Patient or caregiver	Relationship to patient	Age group	Gender	Ethnicity
Line	Patient	—	65+	Female	Niuean
Lote	Patient	—	15–24	Female	Samoan
Masina	Patient	—	55–64	Female	Samoan
Soma	Patient	—	65+	Male	Samoan
Sina	Patient	—	55–64	Female	Niuean
Sala	Caregiver	Daughter	25–34	Female	Samoan
Puna	Caregiver	Mother	45–54	Female	Samoan
Dylan	Caregiver	Father	45–54	Male	Samoan
Tavi	Caregiver	Husband	55–64	Male	Samoan
Leilani	Caregiver	Partner	45–54	Female	Samoan
Kama	Caregiver	Husband	55–64	Male	Samoan

**Figure 1:**
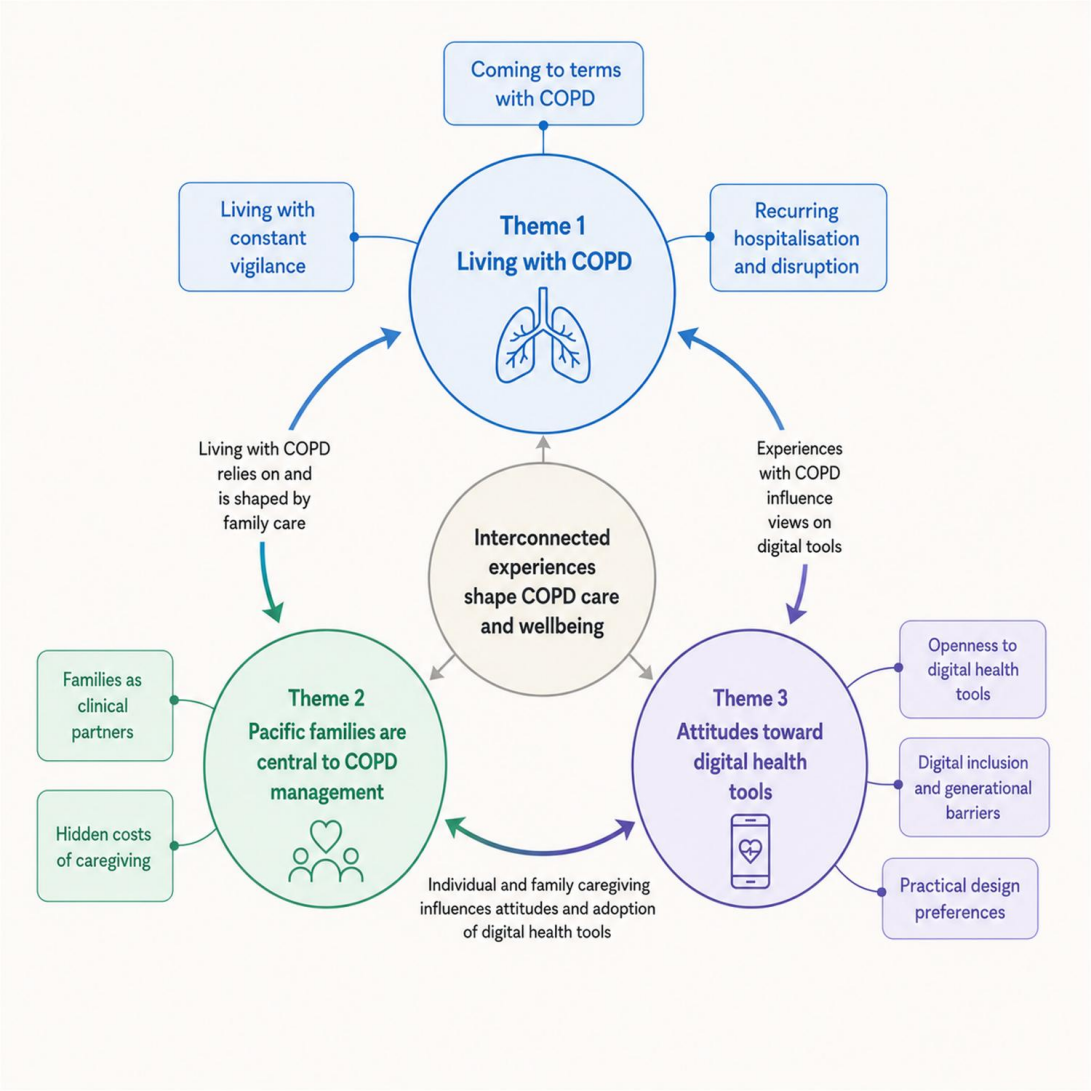
Qualitative themes and subthemes.

The analysis generated three main themes, each with its own subthemes (Figure 1): (1) Living with COPD; (2) Pacific families are central to COPD management, and (3) Attitudes towards digital health tools.

### 3.1 Theme 1: Living With COPD

This theme explored participants' experiences living with COPD and has three subthemes: coming to terms with COPD; living with constant vigilance; and recurring hospitalisation and disruption.

#### 3.1.1 Coming to Terms With COPD

Coming to terms with COPD was a gradual process of adjustment and acceptance for some individuals. When first diagnosed, one participant described holding onto the hope that their condition might resolve on its own or require only minor changes to their lives. Over time however, this gave way to a more grounded understanding of learning to live with COPD:

Part of it in the beginning was being in denial about, you know [COPD] and thinking that it would be okay or something that could be easily resolved. But I think it's just a slight change in lifestyle and learning how to manage it, like recognising the symptoms and…recognising the triggers that would, you know, set me off.

(Masina, living with COPD, Samoan)

For Masina, the process of coming to terms with COPD was complicated by the fact that she had not fully understood their diagnosis in the first place. As she mentions, her diagnosis was discovered not through a direct conversation with a doctor, but through a clinical report seen later. Masina had lived with a condition she was never told about, mistaking it as asthma:

I actually didn't realise that he [doctor] had diagnosed me with COPD. I just thought it was still, you know, just my asthma playing up kind of thing… I didn't realise that he had actually diagnosed me with the condition until I saw it on one of my clinical reports.

(Masina, living with COPD, Samoan)

For a younger participant, COPD carried an emotional weight. The experience of watching others recover while remaining bedridden challenged her assumptions about health and recovery, and the expectation that a young, healthy body should be able to ‘recover faster’:

I thought I was one of those people who could recover faster, being young and being told that, you know…a struggle in itself…watching people just go… and I'm still on the bed…

(Lote, living with COPD, Samoan)

As shared by Soma, he expressed a strong desire for a cure while recognising the irreversible nature of COPD. Regardless of how he feels in the moment, there is recognition that COPD continues to advance once diagnosed:

I wish there is a cure. I wish there is a cure for COPD…deep down I know that the disease is progressing and there is no return once you get told you got COPD.

(Soma, living with COPD, Samoan)

### 3.2 Living with Constant Vigilance

Participants described COPD as a condition that was not simply managed individually, but one that was constantly negotiated as a family collective. Managing COPD at home demanded ongoing vigilance and often led to physical and social isolation:

I can't go out, I can't socialise you know, that life is gone. You're so scared of somebody with perfume, you know, anything smelly. Even the dishwashing liquid, that triggers your COPD.

(Soma, living with COPD, Samoan)

Caregivers often felt responsible for monitoring not only their loved one's condition but also the health of anyone who came into contact with them. Everyday social interactions required careful judgement to reduce risk. As a result, the home was treated as a space that needed to be actively protected and controlled:

I'm very cautious if people want to come over and my dad's feeling, half and half…you know, people laugh, but I'm like, ‘do you have the flu? Have you had any symptoms?’ or anything, because…he's very much vulnerable to catching whatever other virus that's viral in the community.

(Sala, caregiver, Samoan)

For individuals managing their own condition, ongoing self‐monitoring was essential, including the ability to detect early indicators of worsening symptoms and intervene promptly. This proactive approach placed significant responsibility on individuals to detect any decline early and respond quickly:

We came up with a plan that if I do start feeling a bit unwell, then I have to get onto antibiotics straight away just to, you know, so it prevents me getting sicker.

(Masina, living with COPD, Samoan)

Some caregivers had clear response plans worked out with health professionals, giving them a sense of direction during an exacerbation. Understanding what an individual's ‘normal’ health looked like, was important, so any changes in health could be quickly recognised and acted upon:

That's how we manage his COPD and from knowing what his normal looks like, then when there is a shift, what to do. Which you know, everyone that has COPD should have a management plan.

(Sala, caregiver, Samoan)

While it was important to recognise when an individual's health was deteriorating, it was also important to plan for financial disruptions within families. A sudden health emergency disrupted financial and household stability, adding another layer of strain to an already stressful health situation:

I have it in my head what I need to do. I call the ambulance…Financially, I try. I am slowly putting things in place, things that she used to be able to do, I am sort of taking more responsibility of it now.

(Kama, caregiver, Samoan)

### 3.3 Recurring Hospitalisation and Disruption

COPD did not follow a straight path of treatment and recovery for families. Instead, it pulled individuals and families into a repeating cycle of hospital admissions, treatment, discharge, being at home briefly and then being readmitted:

In the last two years he's had repetitive hospital admissions and so last year it was like 7 or 8 admissions last year and they were concurrent months… So, we'd go in then we'd get discharged after a week or a week and a half. Then we'd be out for like two or three weeks and then we'd go back in again.

(Sala, caregiver, Samoan)

As Sala highlights, hospital admissions affected the whole family. Understanding her father's condition and being present during every hospital admission meant navigating the system, monitoring her father's condition, and communicating with health professionals on his behalf. While being an important caregiver and advocate for her father, it also came at the cost of her own physical health:

The back‐to‐back hospital admissions…made me consider my full‐time work because there's not much sick leave you can take and only so much dependent leave you can have to be with your family or your dependents…before you know it, it takes an effect on your job. So, I had to make that decision…but that's how I've pretty much come to becoming his full‐time carer.

(Sala, caregiver, Samoan)

Families learned to adjust and manage the ongoing cycle of hospital admissions for COPD exacerbations with one participant calling the hospital its second home:

I frequently saw the doctors in the early days. Middlemore was my second home…it was almost every month I would go into Middlemore.

(Soma, living with COPD, Samoan)

### 3.4 Theme 2: Pacific Families Are Central to COPD Management

This theme captures the central role Pacific families play in COPD management, not as passive supporters but as clinical monitors, decision‐makers, navigators, and advocates. There are two sub‐themes: families as clinical partners; and hidden costs of caregiving.

#### 3.4.1 Families As Clinical Partners

Family members shared they worked closely with hospital teams and health professionals to develop emergency response plans aimed at helping family members manage their COPD. Importantly, family members took on clinical roles where they tracked vital signs, recognised early deterioration, sourcing information and activating COPD management pathways:

I check his obs [vital signs] daily but it's just knowing what normal looks like, it's important too because me, I think that's the fortunate thing about me being here 24/7, I would notice any slight shift in appearance and the way his breathing is… So that's how we manage his COPD and from knowing what his normal looks like, then when there is a shift, what to do.

(Sala, caregiver, Samoan)

As Sala continues to highlight, it is essential that family members have a management plan for their loved ones:

Everyone that has COPD should have a management plan. So, we have one, we've drafted them up with the respiratory team and our GP… just knowing what to do.

(Sala, caregiver, Samoan)

Working in partnership with healthcare teams enabled families the opportunity to clarify questions around COPD management and unfamiliar terminology early. Clinicians played an active role supporting and guiding families through complex information to build a clearer picture of what their family member was facing:

The people I was talking with were all nurse practitioners and GPs…if I had any questions…they'd ask, what words are you stuck on? What's the terminology that I'm seeing?

(Dylan, caregiver, Samoan)

This was particularly important, as participants noted that developing awareness and understanding about COPD provides a crucial foundation for families as they learn to navigate and manage their condition effectively:

The best thing we can do for our people is education. Education and understanding how COPD works.

(Leilani, caregiver, Samoan)

For our Pacific people it's really raising the awareness… raise awareness and allow and let people know… that it's okay. You know, there are Pacific people that are also going through the journey.

(Tavi, caregiver, Samoan)

#### 3.4.2 Hidden Costs of Caregiving

Several caregivers shared about the mental, social and financial pressures that come with supporting a family member with COPD. One participant referred to the Samoan concept of ‘*tausi matua*’, a cultural expectation that children and extended family will provide care for ageing parents. While this value provided a strong sense of purpose and cultural grounding, it also meant navigating the overlapping pressures of the emotional, social, and financial realities of daily life:

Some days we have good days, some bad days. It affects us physically, mentally and sometimes financially because of some of the things that I need to provide extra.

(Kama, caregiver, Samoan)

The cycle of repeated hospital admissions made being employed increasingly difficult, and eventually impossible for supporting family members. With limited leave entitlements, full‐time work became unsustainable for carers like Sala. The need to remain present for her father during his hospitalisations meant she could no longer be in full time employment:

I'm a full‐time caregiver and I pretty much am in my dad's business, 24/7 really…I take him to the hospital, I stay throughout the whole admission. So, I sleep at the hospital, whether it's on a chair or on the floor, I stay throughout the whole time he's there… that's affected me physically in terms of my own health…it's been quite heavy the last years.

(Sala, caregiver, Samoan)

Of note, caregiving was not always carried alone. Supporting a family member with COPD was a collective effort that required planning, careful coordination and shared responsibilities:

My sister's here on the weekend and on Tuesdays, like she works from here on Tuesdays so that if I have things I need to do during the week, I book everything on the Tuesday while she's working.

(Sala, caregiver, Samoan)

This informal coordination within Pacific families speaks to the collective nature of caregiving, a responsibility that is shared across the family unit, even when one person remains at the centre of it.

#### 3.4.3 Theme 3: Attitudes Toward Digital Health Tools

This theme captures participants' views regarding the use of digital health tools to support managing COPD. It has three sub‐themes: openness to digital health tools; digital inclusion and generational barriers; and practical design preferences.

#### 3.4.4 Openness to Digital Health Tools

There was openness to using digital health tools to help manage COPD by individuals and carers, particularly given the frustration that arises when social interactions become strained because others may not understand the limitations COPD imposes:

I wish people, scientists out there will create something that will cure the COPD and make people breathing better. Because you know going out with some people who don't understand my illness, they get frustrated because I do stop often trying to catch my breath.

(Soma, living with COPD, Samoan)

Several participants were already familiar using a pulse oximeter and had developed a clear, confident understanding of what the readings meant when managing their COPD:

When it's 88%, it's not good…I know my oxygen is low…I know straight away its 84 or 83. Something handy, I put it on and then I know my body is low now. It is important for my kids and husband to know the numbers.

(Line, living with COPD, Niuean)

Rather than relying solely on clinicians to interpret their vital signs, participants were able to recognise when their oxygen saturation levels were within an acceptable range and when they indicated the need for assistance. Having family members understand their numbers and linking this information to their mobile phones was viewed as particularly important, given that phones were always readily accessible:

The only thing I can rely on…is that oximeter. Sometimes it goes down to 87 or 88. You know, that is danger zone…if it's any lower, you just call the ambulance…but it would be nice if there is something on the cell phone because you always have your cell phone next to you.

(Soma, living with COPD, Samoan)

#### 3.4.5 Digital Inclusion and Generational Barriers

While there was strong support for using digital health tools, several participants highlighted significant barriers for elderly Pacific patients. The most consistent concern was accessibility for older adults who were not confident with technology. Participants questioned whether Pacific elders living with COPD would have access to digital devices and, importantly, how they would manage without a caregiver available to provide technological support:

I know you have to take into account that a lot of the older generation that will have the COPD are not tech savvy. Well, my dad, he's 72 compared to his brother that's a bit older than him. My dad doesn't know how to use a touch phone, not even just to make a call.

(Sala, caregiver, Samoan)

Several barriers to using digital health tools were also named including limited English, poor vision or hearing, unaffordable devices, and unfamiliarity with touchscreens:

Something more simple because you know…English is a second language or even if it is in their language, how much can they read, you know, their vision, what is the hearing like or where's their cognition at?' (Sala, caregiver, Samoan)

I think it's really difficult for our elderly people to understand more about apps and stuff. Even myself with an IT background, I do struggle with apps.

(Leilani, caregiver, Samoan)

Of note, a hands‐on device that connected directly to their mobile phone was perceived as more straightforward, particularly when interpreting visual information about their lung function:

I prefer the physical side of things, to actually hold the thing and put it on your nose you know, and you look at the cell phone and see what it's doing in your lungs.

(Soma, living with COPD, Samoan).

#### 3.4.6 Practical Design Preferences

As noted in earlier themes, COPD management was a shared responsibility, that extended beyond the individual to include family members. This recognition shaped how participants thought about technology. Any digital health tool needed to be designed for both the person living with COPD and those caring for them:

When I think about it specifically for my dad… technically I am that monitor and I am the computer that generates whether it's getting worse or better.

(Sala, caregiver, Samoan)

There was a clear preference for a simple, low‐tech device that could be used across different settings. Unlike the CPAP (Continuous Positive Airway Pressure) machine which participants felt was bulky, small wearable or wrist‐based options that was portable and easily integrated into daily routines were favoured:

I think the best thing to have is some small device…you can carry it with you during your exacerbation time. You put it on your finger or a watch.

(Leilani, caregiver, Samoan)

Some portable machines…could be helpful, for people that has COPD. It will just make better quality of life, as they walk around the kitchen, go shopping with it.

(Kama, caregiver, Samoan)

Something handy to test and know what's happening.

(Sina, living with COPD, Niuean)

Importantly, participants stressed that any digital health tool should complement and not replace their own or their carers awareness of their condition and symptoms. Any device used to help assess and manage COPD should be used as prompts or safeguards, rather than a substitute for self‐monitoring:

Would be helpful if they created an app for phones to help track COPD levels or breathing levels, oxygen levels…a good thing for a person to know where they are daily and when to manage what they need.

(Kama, caregiver, Samoan)

Caregivers emphasised that digital health tools must remain reliable and functional during power outages, as this could become a source of stress when respiratory support depended on electricity:

When we have those power cuts, those things could be able to charge, it does not have to depend on the power.

(Kama, caregiver, Samoan)

To assist any new digital health tool being introduced to individuals, a step‐by‐step instruction guide that was translated into Pacific languages, alongside visual aids would support those with limited literacy:

The language on how to use the machine is the thing… you have the options and also have some visuals.

(Kama, caregiver, Samoan)

## 4 Discussion

This qualitative study contributes to the limited research on COPD among Pacific peoples by exploring the experiences of Pacific individuals with COPD and their families in Aotearoa New Zealand. It identified three themes: (1) Living with COPD; (2) Pacific families are central to COPD management, and (3) Attitudes toward digital health tools (Figure 1). The findings demonstrate that these themes are closely interconnected rather than existing as separate concepts. Living with COPD is closely linked to family caregiving, while individual and family caregiving experiences shape attitudes toward digital health tools. Together, these relationships highlight the importance of culturally responsive respiratory services and digital health solutions that support COPD management and wellbeing among Pacific communities.

Participants described coming to terms with a COPD diagnosis as a gradual process of adjustment and described a period of hoping the condition might resolve or prove less serious than it was, before moving toward a more accepted understanding of life with COPD. One participant described not being formally told of her COPD diagnosis and only discovered it later through a clinical report, having long attributed her symptoms to asthma. These experiences echo findings from Indigenous communities in Australia, where Aboriginal peoples with COPD reported inadequate explanations and information at diagnosis, and a strong association between COPD and fear or fatalism [13]. This underscores the importance of clear, timely, and culturally appropriate communication at the point of diagnosis, ideally with family present, so that individuals and their carers can develop a shared understanding of COPD and prepare for managing the condition together over time.

Living with COPD was characterised by a constant state of vigilance, that extended beyond clinical care into everyday life. Participants described continuously monitoring environmental triggers, early warning signs, and physical limits, with exposures such as perfumes or household cleaning products commonly provoking breathlessness. To reduce risk, many withdrew from social settings. Similar experiences have been reported elsewhere, with breathlessness and respiratory symptoms slowing everyday activities and reducing quality of life, often leading individuals to withdraw from social life and events [2, 14]. Meharg et al. [13] observed a parallel dynamic among Aboriginal Australians with COPD, whose participants described how breathlessness and fatigue had limited their capacity to fulfil cultural roles and responsibilities, including supporting family members, participating in community ceremonies, and travelling to Country. These were understood not as incidental lifestyle losses but as profound disruptions to cultural identity and spiritual connection [13].

Considering social connection, family gatherings, and church life are central to Pacific health and wellbeing, avoiding these spaces can create feelings of disconnectedness from key sources of identity, belonging, and support [15, 16, 17]. Pacific communities consistently affirm that places where language, culture, and identity are actively valued and celebrated, make a significant difference to people's sense of self‐worth and wellbeing. Kapeli and colleagues [18] recognise that stronger social support is associated with lower psychological distress among Pacific peoples over time. As such, health for Pacific peoples cannot be understood solely as an individual biological state, but rather a relational condition that exists between people and sustained through ongoing social connections [15, 19]. Losing access to those spaces, even gradually and partially, carries a significant cost to one's wellbeing [16, 17].

Central to this study was the role of family members as active clinical partners and caregivers in COPD management. Family members were involved in daily monitoring, recognising early deterioration, activating emergency plans developed with clinicians, and coordinating care across extended family networks. Living with COPD extends beyond clinical care to ongoing monitoring of triggers, early warning signs, and physical limits. The unpredictable nature of COPD, and repeated hospital admissions reinforced this. Participants described moving frequently between home and hospital, with one referring to the hospital as a ‘second home.’ These cycles of admission had cascading effects on family units, including loss of income, disrupted employment, and caregiver exhaustion, with some family members leaving paid work to provide care.

Within Pacific communities, caregiving responsibilities are underpinned by cultural values that position care as relational, reciprocal and collective [20]. Caring for parents, grandparents and extended kin is understood as a core expression of being Pacific, where the love, care and protection provided by elders are naturally reciprocated by younger generations [20, 21]. This relational ethic, which a participant in this study described as *tausi matua*, places adult children and extended family members at the centre of caregiving. Care is not viewed as an individual task carried by a single caregiver, but as a shared responsibility distributed across the family network, with roles shaped by kinship ties, reciprocity and collective support [20, 21].

Despite the critical role of family members as caregivers, much of this labour remains largely invisible within New Zealand's health system and carries substantial personal and economic costs. National data indicates that one in seven adult New Zealanders is an unpaid carer, with Māori and Pacific peoples disproportionately represented [22]. Caregiving responsibilities commonly span medication management, attending appointments, preparing culturally appropriate meals, providing transport and interpretation, and offering emotional and spiritual support – roles comparable to those of a funded healthcare workforce. The estimated economic value of this care exceeds $17.6 billion annually, yet it remains largely unrecognised within formal health planning [22].

Participants in this study expressed genuine openness and motivation to using digital health technologies for monitoring COPD, particularly devices that could provide real‐time feedback on oxygen saturation and link directly to personal and family members' mobile phones. Several participants were already using pulse oximeters with confidence, interpreting their own readings and using it to manage their COPD symptoms. This aligns with findings from Mirza et al. [23], who explored Pacific perspectives on AI and digital technology for asthma management in Aotearoa New Zealand, the closest comparable study to this present work. The study found that Pacific participants were generally open to digital health tools, particularly where tools could generate early warnings, support family monitoring, and be shared across the family. One participant in Mirza's study described the need for technology to be designed as a ‘family tool’, where the whole family could be alerted and prepared when symptoms worsened, which resonated deeply with Pacific values of collective support and mutual responsibility [23].

While promising, the generational digital divide was articulated clearly by participants. Considering they are the very group most likely to have COPD and have English as a second language, they were identified as least able to use touchscreen devices, often relying on younger family members to set up basic technology. Cost of devices, internet access, and digital literacy were named as barriers to adopting digital health tools. These findings are consistent with studies which have similarly found that older Pacific communities require substantially more support than younger, more digitally literate family members, and recommend that any digital health tool designed for Pacific communities need to be simple, affordable, operable offline, supported by multilingual instructions and include family members [23, 24, 25]. These barriers reflect a persistent global pattern, whereby those who might benefit most from digital health interventions face the greatest obstacles to accessing them. Across Indigenous and First Nations communities, digital health tools have repeatedly failed to reach the most underserved populations due to socioeconomic disadvantage, digital exclusion, and the lack of culturally safe co‐design [8, 26].

Advances in digital health technologies, such as wearable devices and mobile health applications, have been shown to improve the management of COPD [27]. The findings of this study emphasise the importance of simple design features such as visual supports, Pacific language resources, and reliable functionality during power outages. Importantly, participants were clear that digital health tools should support, rather than replace, existing self‐management practices and family‐based monitoring. Park and authors (2025) emphasise that digital tools are most effective when embedded within existing care pathways and designed to complement self‐management and clinician oversight rather than operate as standalone solutions. If digital health tools are not deliberately designed for accessibility and inclusion, they may inadvertently widen inequities among already marginalised communities [8, 27].

Overall, this study demonstrates that living with COPD for Pacific peoples is a collective experience, shaped by family and relational obligations that must be central to care responses. Accordingly, digital health tools designed to support COPD management should align with Pacific values, reinforce family‐based care, and integrate seamlessly within existing clinical and community care pathways.

### 4.1 Strengths and Limitations

A key strength of this study is its contribution to the small but growing body of research on COPD among Pacific peoples. The use of the *talanoa* methodology created a culturally safe and relational context for participants to share experiences that many had not previously discussed. The inclusion of both individuals with COPD and family caregivers also provided a fuller picture of their COPD experiences than patient‐only studies typically achieve.

Limitations include the relatively small sample, the Auckland‐based participants, and the predominance of Samoan participants, which may limit transferability to other Pacific ethnic groups and geographic contexts. Additionally, this study captured experiences at a single point in time and did not follow participants through episodes of exacerbation or hospitalisation. Future research should include a broader range of Pacific ethnic groups, seek participants from outside Auckland, and explore longitudinal experiences, including the impacts of specific clinical interventions on Pacific COPD outcomes.

## 5 Conclusion

This study explored the lived experiences of Pacific peoples living with COPD and the perspectives of their family caregivers in Aotearoa New Zealand, highlighting that COPD is experienced not as an individual condition but as a collective and relational journey. Family members play central roles as caregivers and clinical partners, absorbing significant emotional, social, and economic impacts. Participants expressed openness to digital health tools for COPD management, provided these technologies are culturally aligned, family‐inclusive, and integrated into existing care pathways. Together, these findings highlight the need for COPD services and digital health interventions that are culturally grounded, relational, and equity‐focused to better support Pacific peoples living with COPD. The clarity and consistency of these findings provide a strong foundation for larger, focused studies to further examine family‐centred COPD care and the co‐design of digital health tools aligned with Pacific values.

## Author Contributions

**Amio Matenga‐Ikihele:** methodology, writing – original draft, writing – review and editing, formal analysis, investigation. **Joshua Ali'ifa'alogo:** methodology, writing – original draft, writing – review and editing, formal analysis, investigation. **Michaela Roberts:** methodology, writing – review and editing, investigation. **Amy Chan:** conceptualisation, writing – review and editing. **J. Geoffrey Chase:** funding acquisition, writing – review and editing, conceptualisation. **Ella F.S. Guy:** funding acquisition, writing – review and editing, conceptualisation.

## Conflicts of Interest

The authors declare no conflicts of interest.

## Data Availability

The data that support the findings of this study are available on request from the corresponding author. The data are not publicly available due to privacy or ethical restrictions.
